# Selenium Compounds, Apoptosis and Other Types of Cell Death: An Overview for Cancer Therapy

**DOI:** 10.3390/ijms13089649

**Published:** 2012-08-02

**Authors:** Carmen Sanmartín, Daniel Plano, Arun K. Sharma, Juan Antonio Palop

**Affiliations:** 1 Department of Organic and Pharmaceutical Chemistry, University of Navarra, Irunlarrea 1, Pamplona E-31008, Spain; E-Mails: dplano@alumni.unav.es (D.P.); jpalop@unav.es (J.A.P.); 2 Department of Pharmacology, Penn State Hershey Cancer Institute, Penn State Hershey College of Medicine, CH72, 500 University Drive, Hershey, PA 17033, USA; E-Mail: aks14@psu.edu

**Keywords:** apoptosis, autophagy, cell death, selenium

## Abstract

Selenium (Se) is an essential trace element involved in different physiological functions of the human body and plays a role in cancer prevention and treatment. Induction of apoptosis is considered an important cellular event that can account for the cancer preventive effects of Se. The mechanisms of Se-induced apoptosis are associated with the chemical forms of Se and their metabolism as well as the type of cancer studied. So, some selenocompounds, such as SeO_2_ involve the activation of caspase-3 while sodium selenite induces apoptosis in the absence of the activation of caspases. Modulation of mitochondrial functions has been reported to play a key role in the regulation of apoptosis and also to be one of the targets of Se compounds. Other mechanisms for apoptosis induction are the modulation of glutathione and reactive oxygen species levels, which may function as intracellular messengers to regulate signaling pathways, or the regulation of kinase, among others. Emerging evidence indicates the overlaps between the apoptosis and other types of cell death such as autophagy. In this review we report different processes of cell death induced by Se compounds in cancer treatment and prevention.

## 1. Introduction

**Figure 1:**
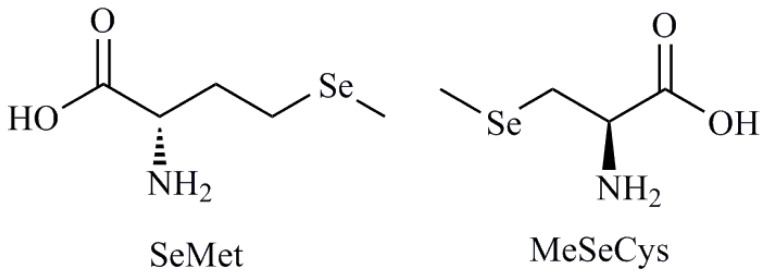
Selenomethionine and methylselenocysteine.

Selenium (Se) is an oligoelement with essential biological functions and belongs to the most extensively studied chemopreventive compounds [1,2]. Foods (cereals, grains, vegetables) contain diverse amounts and chemical forms of Se [3]. An adequate Se intake has been estimated at 50 μg/day with toxic levels being estimated to occur with intakes of the order of 350–700 μg/day [4]. Se is important for many cellular processes, because it is a component of several selenoproteins with preventive function of some forms of cancer [5,6]. Se supplementation with low doses seems to be beneficial not only for cancer prevention, but it can positively influence many other functions in an organism by reducing inflammations, heart diseases and regulating the blood pressure [7]. Effectiveness of Se compounds as chemopreventive agents *in vivo* is correlated with their abilities to effect the regulation of the cell cycle, to stimulate apoptosis and to inhibit tumor cell migration and invasion *in vitro* [8]. The cell cycle is a conserved mechanism by which eukaryotic cells replicate themselves. The eukaryotic cell cycle is divided into four major phases as follows: the G_1_ phase before DNA replication, the periods of DNA synthesis (S phase), the G_2_ phase before cell division and cell division (M phase). On the other hand, apoptosis is also a highly conserved mechanism by which eukaryotic cells commit suicide and enable an organism to eliminate unwanted and defective cells during normal development, turnover and pathological conditions. In humans and animals, cell proliferation and death must be regulated to maintain tissue homeostasis. Apoptosis induced by supranutritional doses of this compound was described in various types of neoplastic cells, including prostate, colon and liver cancer, leukemia and lymphoma [7]. There are several proposed mechanisms to explain the effect of Se on cell cycle and apoptosis and it has been well recognized that Se plays a key role in these processes but mechanisms for Se action are very complex and not fully understood. They involve protein kinases signaling, activation of caspases, p53 phosphorylation and reactive oxygen species (ROS) generation [9–14]. Besides, it is widely recognized that the effectiveness of Se compounds as chemopreventive and anticancer agents is correlated with their chemical form and doses. The chemical structure dependence is exemplified in the contradictory results of two clinical trials using 200 μg of Se supplementation per day in the prevention of cancer. In the Nutritional Prevention of Cancer (NPC) Trial, supplementation of Se enriched yeast was shown to reduce the incidence of a number of cancers [15] including prostate cancer [16], whereas the Se and Vitamin E Cancer Prevention Trial (SELECT), that used selenomethionine (SeMet) (Figure 1), major component of Se yeast, ceased early as there was no reduction in the incidence of prostate cancer [17]. Therefore, other components of Se yeast, e.g., methylselenocysteine (MeSeCys) may be contributing to the overall efficacy of Se yeast used in NPC trial. So, two similar compounds, *i.e.*, MeSeCys and SeMet can have disparate efficacies as anticancer agents. The reason behind this fact is the difference in the speciation of Se in cells treated with MeSeCys or SeMet, mainly in the potential redox-altering capabilities of the metabolites of MeSeCys as evidenced by the presence of diselenides [18]. Tapiero *et al.* [19] have reported that metabolites of Se compounds have been shown to induce ROS, which in turn can induce oxidative modifications and DNA stand breaks. Studies have focused on Se-induced stress responses in various cultured cancer cells, from which it is suggested that much of the role of Se in cancer prevention is attributable to ROS-induced apoptosis or cell cycle arrest in cancer cells. Consistent with this notion, it has been shown that Se-induced apoptosis in cancer cells can be suppressed by antioxidants and is p53-dependent. Furthermore, Se can sensitize cancer cells to other apoptotic inducers, including tumor necrosis factor-related apoptosis-inducing ligand (TRAIL) and doxorubicin. The observation that Se specifically induces senescence response in non-cancerous cells suggests a cost-effective scenario by which tumorigenesis can be stifled at the very beginning in individuals who consume Se with a cancer prevention perspective. In addition, prior to occurrence of apoptosis, Se compounds alter the expression and/or activities of signaling molecules, mitochondria associated factors, transcriptional factors, tumor suppressor genes and cellular reduced glutathione. Among the metabolites of Se derivatives it was demonstrated that methylselenol metabolite pool has many desirable attributes of chemoprevention whereas the hydrogenselenide pool with excess of selenoprotein synthesis can lead to DNA single-strand breaks [20].

On the other hand, there are emerging evidences that cell death caused by Se and Se compounds can occur not only by apoptotic pathway but can also proceed by non-apoptotic modes such as autophagy, necrosis, mitotic catastrophe or combinations thereof, because there are overlaps among them [21]. This has been demonstrated in NB4 cells treated with sodium selenite that suppressed autophagy and increased apoptosis through phosphoinositide-3-kinase (PI3K)/Akt [22]. In addition, Suzuki *et al.* [23] have examined the contribution of autophagy to the mechanism of apoptosis caused by SeMet in A549 lung carcinoma cells. They demonstrated that the Akt/mammalian target of rapamycin (mTOR)/ROS pathway was affected but no evidences were found for autophagy. This article covers recent reports on various processes by which Se compounds cause cancer cell death. A classification of Se compounds discussed in this review article is presented in Table 1.

## 2. Apoptosis and Kinases Modulation

**Figure 2:**
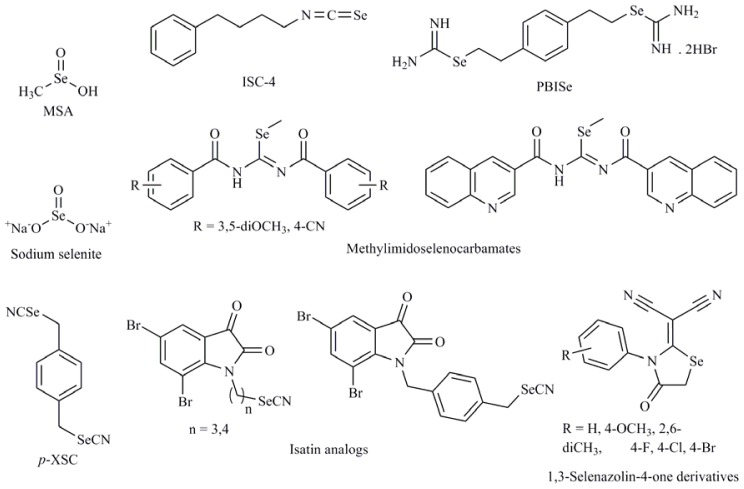
Sodium selenite, 1-4-phenylenebis(methylene)selenocyanate (*p*-XSC), 4-phenylbutylisoselenocyanate (ISC-4), 3,5-dimethoxy and 4-cyanophenyl imidoselenocarbamates, quinolinimidoselenocarbamate (EI201), 1,3-selenazolin-4-one, (1,2-ethanediyl)bisisoselenourea (PBISe), methylselenic acid.

Recent reports have shown that Se compounds are able to induce tumor cell apoptosis through distinct mechanisms according to cell type and compound pattern. It is known that several protein kinase pathways play essential roles in mediating mitogenic and antiapoptotic signals and can regulate cell proliferation and survival. The mechanism of apoptosis induction of Se compounds is variable depending on their structure, metabolism and type of cell line studied. So, methylselenol, produced by metabolism of SeMet inhibits extracellular signal-regulated kinase (ERK1/2) pathway activation in fibrosarcoma tumor cells HT1080 [24]. However, tumor inhibition by sodium selenite (Figure 2) in human colon cancer cell lines HCT116 and SW620 is associated with activation of c-Jun NH_2_ terminal kinase (JNK) [25]. The PI3K pathway mediates cell cycle progression and cell survival through downstream effector proteins, one of which is the protein kinase Akt. It is widely accepted that Akt plays a crucial role in controlling cell survival and apoptosis in cancer cells. It is activated by insulin or growth factors and promotes cell survival by inactivating multiple targets including Bad, caspase-9, and forkhead transcription factors. There is a growing body of evidence describing its role in promoting cell transformation and tumorigenesis. 1,4-phenylenebis(methylene)selenocyanate (*p*-XSC) (Figure 2) induced apoptosis in androgen responsive LNCaP and androgen-independent LNCaP C4-2 human prostate cancer cells by decreasing Akt phosphorylation [26]. The Akt3 signaling pathway plays a central role in deregulating apoptosis to promote development of approximately 70% of melanomas. Nguyen *et al.* [27] have reported a structurally related compound, 4-phenylbutylisoselenocyanate (ISC-4) (Figure 2) that decreased Akt3 signaling and led to a 3-fold increase in apoptosis rates. Later, in a nude mouse model injected with wild-type HT-29 colon cancer cells, ISC-4 was shown to inhibit tumor growth significantly via the inhibition of Akt, which was more pronounced in culture cells than others Akt inhibitors [28]. There have been other reports showing Se compounds with selenocyanate moiety to act as Akt modulators. For example, some isatin analogs (Figure 2) containing a selenocyanate group in the alkyl chain may be promising compounds induced apoptosis in breast cancer cells, accompanied of inhibition of tubulin polymerization and a light effect in Akt phosphorylation [29]. Other chemical forms of Se, such as sodium selenite, are able to downregulate β-catenin signaling pathway through inhibiting Akt kinase in colorectal cancer cells and colon xenograft tumors. Selenite could induce apoptosis in colorectal cancer cells through inhibiting Akt/β-catenin survival axis in a ROS-dependent manner [30]. Recently, our research group has described that the 3,5-dimethoxyphenyl and 4-cyanophenyl methylseleno imidocarbamates (Figure 2) in the prostate cancer cell (PC-3) caused an important inhibitory effect in Akt and ERK phosphorylation, two key nodes in PI3K and mitogen activated protein kinase (MAPK) pathways, respectively. Moreover, these compounds also inhibited the action on both mTORC1 and mTORC2, implicated in PI3K and MAPK pathways, through the dephosphorylation p70S6K and 4E-BP1 as well as Akt on Ser473 in PC-3 cells, thus, emerging as promising inhibitors of the PI3K/Akt/mTOR and MAPK pathways [31]. In addition, other quinolinimidoselenocarbamate (EI201) [32] (Figure 2) exhibited a strong antitumor activity by multi kinase inhibition with aberrant upregulation of PI3K/Akt and MAPK signaling pathways.

In order to improve the safety for Se compounds, the formulation as nanoparticles is an interesting approximation. The encapsulation of Se at nanoparticle size (Nano-Se) upregulates selenoenzyme activity efficiently with less toxicity compared with other seleno-compounds. A study carried out in LNCaP cancer cells with Nano-Se suggested that Akt catalyzed androgen receptor (AR) phosphorylation was a key step for AR ubiquitination by Mdm2 and subsequent degradation by 26S proteasome. AR ubiquitination was significantly impaired in Mdm2-deficient mouse embryonic fibroblasts (MEFs). It was found in this study that Nano-Se activated Akt by promoting Akt phosphorylation at Ser473 and Thr308, which started the downstream AR degradation cascade, including phosphorylation and activation of Mdm2, and phosphorylation and polyubiquitination of AR. When they used LY294002 to block the PI3K/Akt pathway, the Akt phosphorylation induced by Nano-Se was almost disappeared, and Akt regulated Mdm2 phosphorylation was missing too. Furthermore, Nano-Se inhibited prostate cancer cell growth partially by caspase-mediated apoptosis, which was through the downregulation of AR expression at both transcriptional and translational levels. Nano-Se treatment activated the Akt/Mdm2 pathway, and initiated AR phosphorylation, ubiquitination and degradation. The cancer suppression function of Nano-Se consisted of at least two mechanisms, regulation of AR transcription and promotion of AR protein degradation [33].

Among the targets for seleno derivatives are the MAPKs. Several members of the MAPK family have been identified, including ERK, SAPK/JNK and p38 MAP kinase. Many subsequent studies have confirmed an anti-apoptotic role for ERK1/2 and a pro-apoptotic role for sustained activation of p38 and JNK. For example, the 1,3-selenazolin-4-one derivatives (Figure 2) induced apoptosis in prostate cancer cells (PC-12) through ERK1/2 phosphorylation *via* MEK activation [34]. Sodium selenite treatment in a male Balb/c mice model resulted in increased expression of p38 and JNK MAPK, decreased Bcl-2 expression, and increase in caspase-3 expression [35,36]. The Se,Se′-1,4-phenylenebis (1,2-ethanediyl)bisisoselenourea (PBISe) (Figure 2) used for topical application retarded melanocytic lesion development due to decreased Akt3 signaling, which increased MAP kinase pathway activity leading to decreased cell proliferation and increased apoptotic cell death [37]. Cyclin-dependent kinases (CDKs) govern the transition between phases during cell cycle progression and therapeutic strategies that block CDK activity are therefore unlikely to target tumor cells selectively. It has also been seen that in prostate cancer cells (DU-145), MSA acts by upregulation of CDK inhibitors (CDKIs) including p16/INK4a, p21/CIP1 and p27/KIP1. These CDKIs bind with their respective cyclin/CDK complexes and inhibit the kinase activities of CDK4, CDK6 and CDK2 [38]. One of the targets for sodium selenite is protein kinase Cα (PKCα), which is involved in both tumor promotion and progression. Moreover, several of the tumor promotion or cancer prevention mechanisms may involve redox-sensitive targets. This protein played an antiapoptotic role through its effects on ERK1/2 and Akt in a study carried out in human leukemia NB4 cells [39].

## 3. Apoptosis and Caspases

**Figure 3:**
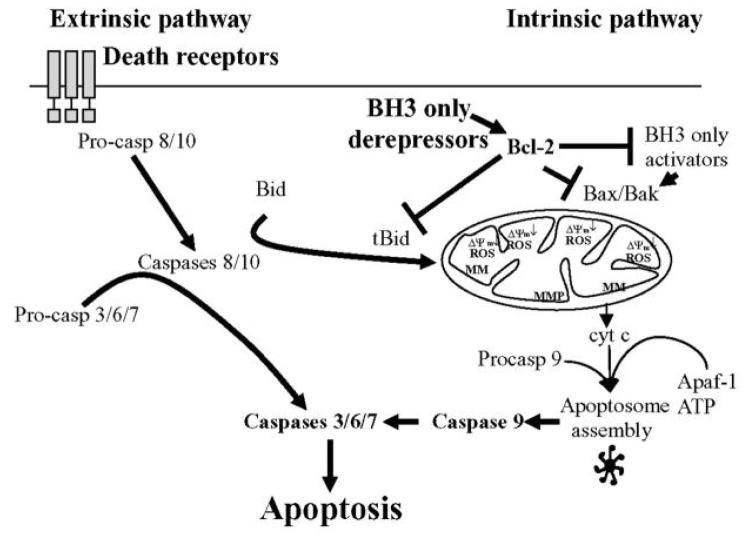
Simplified diagram of molecular pathways that regulate caspase-dependent apoptotic cell death.

The caspases are a family of cysteine proteases whose main function is the central regulation of cell death. Of the 12 caspases that have been identified in humans, 7 transduce apoptotic signals during programmed cell death. All caspases are expressed as inactive zymogens (procaspases) and share key structural features: an NH_2_-terminal pro-domain of variable length is followed by a catalytic domain consisting of a large (17–20 kDa) subunit and a small (10–12 kDa) subunit. Caspases strongly overlap in specificity, and apart from the critical amino acid residues that bind into the catalytic cleft also flanking amino acids as well as the tertiary/quaternary structure of the substrate determine the efficiency of its cleavage. Caspases can be classified into initiator, such as caspase-2, -8, -9 and -10, and executioner caspases, such as caspase-3 and -7. Initiator caspases have a long N-terminal prodomain, which mediates the formation of protein complexes that provide the molecular platform for caspase activation and inhibition. Initiator caspases cleave a few specific substrates, including executioner caspase zymogens. This cleavage activates the executioner caspases, which in turn cleave their respective substrates, eliciting apoptotic cell death, with its characteristic morphological features of membrane blebbing, pyknotic nuclei, cell rounding and apoptotic vesicle formation. Caspase activation can be regulated through an extrinsic or intrinsic signaling pathway. The extrinsic pathway, which involves Fas and tumor necrosis factor receptors (TNFRs) stimulation, activates caspase-8. The intrinsic pathway, which may be the primary means of activating apoptotic caspase in mammals, triggers the mitochondrial release of cytochrome c (cyt-c), which oligomerizes with Apaf-1 and procaspase-9 to form the apoptosome complex. Activated caspase-9 in this complex activates caspase-3 to execute apoptosis [40–42] (Figure 3).

**Figure 4:**
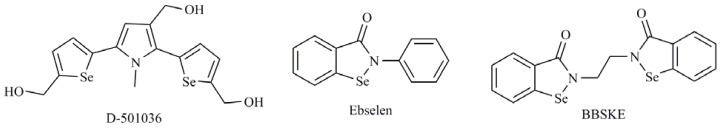
1,2-[Bis(1,2-benzisoselenazolone-3-(2*H*)-ketone)]ethane (BBSKE), Ebselen and 2,5-bis(5-hydroxymethyl-2-selenienyl)-3-hydroxymethyl-*N*-methylpyrrole (D-501036).

Among currently identified Se compounds that possess caspase modulation activity, heterocycles containing Se represent an important class of small molecules useful in cancer chemotherapy. 1,2-[Bis(1,2-benzisoselenazolone-3-(2*H*)-ketone)]ethane (BBSKE) (Figure 4), which is structurally related to Ebselen (Figure 4), is a thioredoxin reductase inhibitor that has demonstrated inhibition in the growth on the tongue cancer Tca8113 promoting the activity of caspase-3 [43]. Another interesting compound with broad spectrum antitumor activity is 2,5-bis(5-hydroxymethyl-2-selenienyl)-3-hydroxymethyl-*N*-methylpyrrole (D-501036) (Figure 4) [44]. D-501036 is an apoptotic inducer through an increase of the activities of caspase-9 and -3 in a dose and time dependent manner.

**Figure 5:**
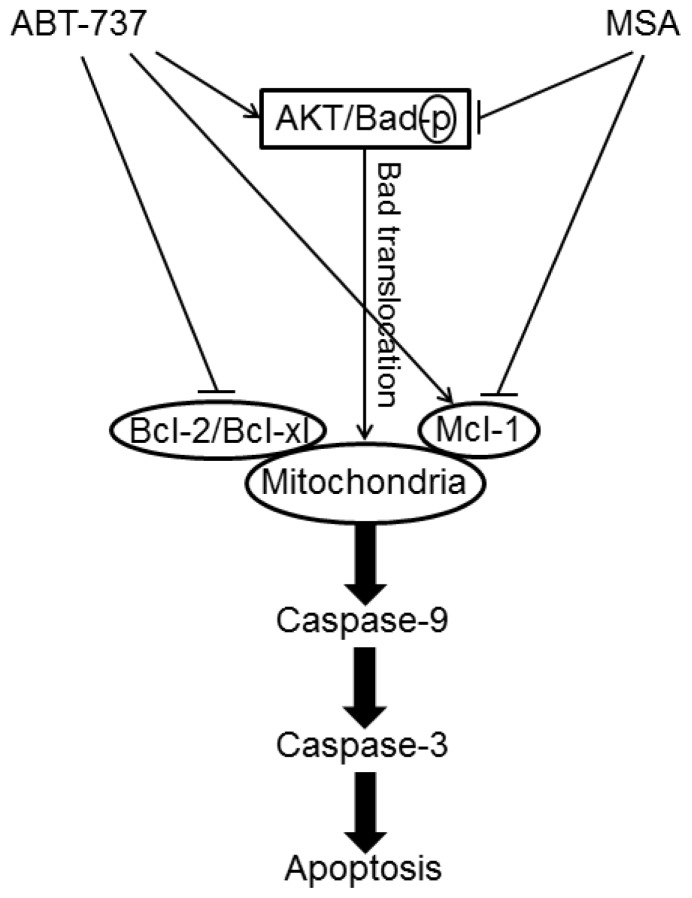
Signaling pathways underlying ABT-737 and/or Methylseleninic acid (MSA) induced apoptosis in cancer cells.

Methylseleninic acid (MSA) has been shown to target caspase modulation both alone and in combination with other anticancer agents. Li *et al.* [45] have demonstrated that MSA acts synergistically when combined with tamoxifen in both tamoxifen-sensitive and tamoxifen-resistant breast cancer cells through detailed analysis of apoptotic pathways, mainly by sequential activation of caspase-9 and then caspase-8. More recently, this practical combination approach was applied to improve the efficacy of chemotherapeutic agents, such as ABT-737, a novel small molecule inhibitor of Bcl-2 family proteins. MSA acted synergistically with ABT-737 to sensitize aggressive cancer cells of human breast (MDA-MB-231), human colon (HT-29) and human prostate (PC-3) to apoptosis induction through enhanced caspase-8/-9/-3 activation [46] (Figure 5).

Similar results have been obtained by combination of MSA with paclitaxel for the treatment of triple negative breast cancer and mainly the synergism was attributable to more pronounced induction of caspase mediated apoptosis [47]. Sodium selenite is another widely studied Se derivative in cancer therapy. Selenite has been reported to induce cell death in several types of cells via the mitochondrial pathway by a combination of indirect or direct effects with varying contribution of caspases. In cervical carcinoma cells, selenite induced caspase-independent apoptosis [48]. When combined with docetaxel in prostate cancer cells (PC-3) it significantly increased the proapoptotic factor caspase-3 but not with docetaxel alone in monotherapy [49]. These results suggest that the mechanism of apoptotic induction may differ between the two cell lines.

**Figure 6:**
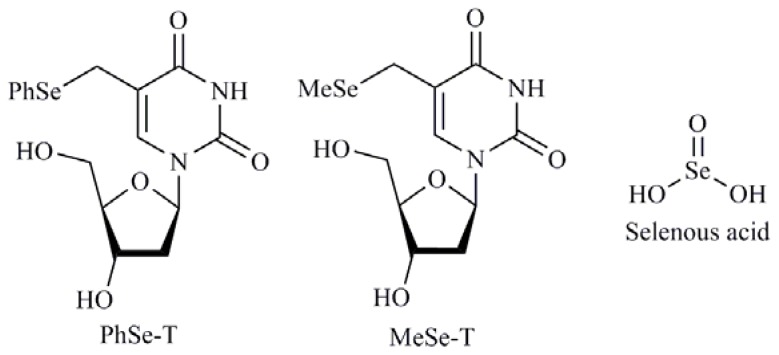
5-Phenylselenyl-methyl-2′-deoxyuridine (PhSe-T) and 5-methylselenyl-methyl-2′-deoxyuridine (MeSe-T), Selenous acid.

A major class of chemotherapeutic agents used in the treatment of cancer are nucleoside derivatives, synthetic compounds that are structurally similar to natural nucleosides. The cytotoxic nucleoside derivatives used in cancer chemotherapy, including cytarabine, cladribine, fludarabine, and gemcitabine, are phosphorylated by intracellular nucleoside and nucleotide kinases to produce the pharmacologically active form related DNA-damaging thymidine nucleoside derivatives. Taking in consideration these structures Kim *et al.* [50] reported the synthesis and cytotoxic effects of 5-phenylselenyl-methyl-2′-deoxyuridine (PhSe-T) and 5-methylselenyl-methyl-2′-deoxyuridine (MeSe-T) (Figure 6), as possible new agents for the treatment of human cancer and to elucidate the signaling pathway that mediates nucleoside derivatives induced apoptosis in human cancer cells. These compounds exhibited apoptotic effects involving caspase-2 and -3 and, to a lesser extent, caspase-8 and -9. Later, the same authors published a study in HL-60 cells where they suggested that the apoptosis induction by PhSe-T and MeSe-T is mediated by the p38 pathway that served as a link between ROS generation and DNA damage/caspase activation [51]. Besides, selenous acid (Figure 6) and sodium selenite in combination treatment with 5-fluorouracil (5-FU) are able to induce apoptosis in certain colorectal cancer cells such as RKO by activation of caspase-8 and -9 [52].

## 4. Apoptosis and Reactive Oxygen Species

Reactive oxygen species (ROS), such as superoxide anion (O_2_^−^), hydrogen peroxide (H_2_O_2_), and hydroxyl radical (HO·), consist of radical and non-radical oxygen species formed by the partial reduction of oxygen. Cellular ROS are generated endogenously as in the process of mitochondrial oxidative phosphorylation, or they may arise from interactions with exogenous sources such as xenobiotic compounds. When ROS overwhelm the cellular antioxidant defense system, either through an increase in ROS levels or a decrease in the cellular antioxidant capacity, oxidative stress occurs. Oxidative stress results in direct or indirect ROS-mediated damage of nucleic acids and has also been implicated in the inhibition of DNA repair. Not only the excess in ROS levels has the potential to contribute to oncogenesis, but high levels of ROS also persist in tumor cells, and can contribute to reduced susceptibility to apoptosis. The presence of excess ROS in cancer cells means that tumors often exist under mildly oxidative conditions. Because of this heightened basal level, the cancer cells become more susceptible to further oxidative stress than normal cells because their endogenous antioxidant systems can be overwhelmed. This important biological difference between normal and neoplastic cells may potentially be exploited therapeutically by agents that further augment ROS levels or weaken antioxidant defenses in cancer cells. The vulnerabilities of cancer cells to oxidative signals are now recognized as a potential target for the rational design of new anticancer agents. The overall cellular redox state is regulated by three systems, two of which are dependent on glutathione: the reduced glutathione (GSH)/oxidized glutathione (GSSG) system; the glutaredoxin (Grx) system; and the thioredoxin (Trx)/Trx reductase (TR) system [53,54].

### 4.1. Apoptosis and Glutathione System

**Figure 7:**
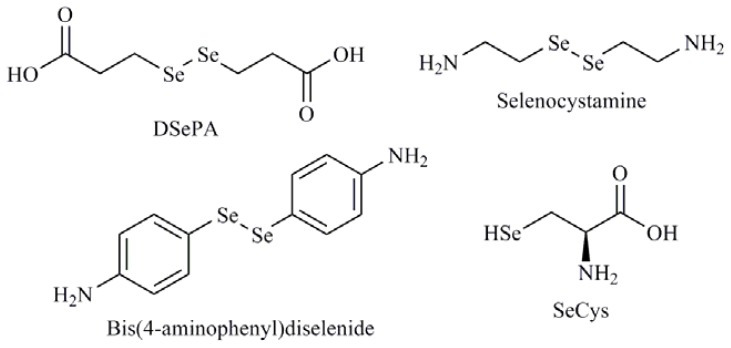
Selenocystamine, diselenodipropionic acid (DSePA), bis(4-aminophenyl)diselenide, selenocysteine (SeCys).

Many of the Se agents in development that modulate redox status or mediate their effects are inorganic derivatives. So, the treatment of human oral squamous carcinoma cells (HSC-3) with selenium dioxide (SeO_2_) and sodium selenite induced apoptosis with possible role of glutathione [55]. Furthermore, sodium selenite caused cytotoxicity in fish by inducing oxidative stress. The cellular lipid peroxidation tends to enhance with increasing selenite exposure dose leading to increase of cell death associated with caspase-3/7 activity [56]. A similar behavior was observed with SeMet in the same biological model [57]. On the other hand, Chen *et al.* [58] in a study with three compounds *i.e.*, sodium selenite, selenocystamine and diselenodipropionic acid (DSePA) (Figure 7), observed provoked oxidation of glutathione with superoxide generation suggesting that the catalytic species producing superoxide were the GSSe^−^ or RSe^−^ anion. In 2010, our research group described the biological evaluation of a library of 59 organoselenium compounds as superoxide generators. Among them, the compound bis(4-aminophenyl)diselenide (Figure 7) showed apoptogenic effect mediated by ROS in lymphocytic leukemia cells (CCRF-CEM) [59]. Later, Kunwar *et al.* [60] demonstrated that DSePA exhibited protective effects in albino mice exposed to whole body gamma radiation, implicating the maintenance of antioxidant enzymes to be the mechanism of action. Sodium selenite and MSA have also been shown to induce ROS in human high metastatic large cell lung cancer cell line L9981 [61]. These same authors have published the potential of MSA as an antioxidant which scavenged ROS and maintained the stability of redox status in two human lung cancer cell lines, L9981 and 95D, showing anticancer effect and apoptosis detection [62]. Other relevant mechanism implicated in the control of the redox balance is mediated, to a large extent, by selenoproteins that contain Se in form of selenocysteine (SeCys) (Figure 7) within their active site, which might be directly responsible for apoptosis induction. [63].

### 4.2. Apoptosis and Thioredoxin System

**Figure 8:**
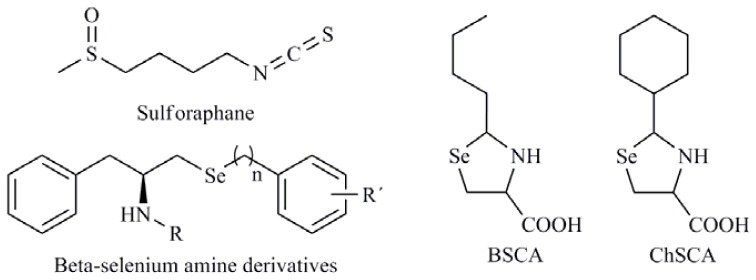
2-Buthylselenazolidine-4-(*R*)-carboxylic acid (BSCA), 2-cyclohexylselenazolidine-4-(*R*)-carboxylic acid (ChSCa), beta-selenium amine derivatives, sulforaphane.

Thioredoxin reductase (TR) is a selenoprotein that functions to reduce the oxidoreductase thioredoxin (Trx) in a NADPH-independent manner. This Trx system is an important regulator of cellular redox status. TR is also involved in cell proliferation, DNA replication, cell cycle, transcription factor regulation, and other redox-sensitive cell signaling pathways. TR contains Se in the form of selenocysteine as the penultimate residue at the C-terminus. In humans, TR is found in all tissues and is expressed as two major isoforms: cytosolic (Trx-1) and mitochondrial. Trx-1 is overexpressed in many human tumors and it is associated with aggressive tumor growth and decreased patient survival. Poerschke *et al.* [64] studied the behavior of six seleno derivatives, 2-butylselenazolidine-4-(*R*)-carboxylic acid (BSCA), 2-cyclohexylselenazolidine-4-(*R*)-carboxylic acid (ChSCA) (Figure 8), SeCys, MSA, *p*-XSC and SeMet, in lung cancer cells (A549) and observed that Trx-1 modulated the cytotoxic effects of BSCA, ChSCA and SeCys by modulation of mitochondrial dysfunction. Similar effects were observed in colon cancer cells (RKO) with SeMet, *p*-XSC, and MSA, particularly for the last one. MSA displayed increased cytotoxicity when Trx-1 levels were reduced [65]. Taking the aforementioned into account, Trx-1 is considered to be an important anticancer drug target and to be involved in both carcinogenesis and cancer progression. Recently, ethaselen (before BBSKE) was identified as the first Se-containing inhibitor of mammalian Trx-1 [66]. Moreover, the addition of Se, for example as selenite, can enhance the cell sensitivity toward some chemotherapeutic agents, such as acylfulvenes or illudin S by inhibition of Trx-1 [67]. Mammalian TR isoforms have broad substrate specificity, reducing not only thioredoxins but also inorganic and organic Se compounds. Therefore, Se compounds can be substrates of TR and the introduction of a substituent group in a non-substituted Se molecule can modify its biochemical and pharmacological properties, including its interaction with thiols and its reduction by TR. So, de Souza *et al.* [68] evaluated the potential influence of different radical substituent on the antioxidant capacity of *β*-selenium amine compounds (Figure 8) and how these structural modifications could influence in their activities functioning as synthetic mimetic of antioxidant enzymes of mammalian organisms. Gundimeda *et al.* [69] have also suggested that the employment of Se compounds, for example MSA, with agents that target TRx-1 could increase the effectiveness or decrease the resistant on malignant cells [70]. Moreover, interdependence between the Se status of the selenoprotein GPx2 and sulforaphane (SFN) (Figure 8) was observed in experimental colon cancer indicating that both of them may act cooperatively [71].

## 5. Apoptosis and p53 Regulation

The p53 protein is a homotetrameric transcription factor that regulates expression of a wide variety of genes through direct binding to response elements in DNA. The best understood function of p53 is to respond to an array of cellular stresses, such as DNA damage, oncogene expression, nucleotide depletion, and aberrant growth signals by inducing cell cycle arrest, DNA repair, differentiation, senescence, or apoptosis. The p53 protein senses and integrates these various stresses via a panoply of post-translational modifications, including phosphorylation, acetylation, and ubiquitination. p53 protein is expressed constitutively, but levels are normally kept low by its rapid ubiquitination by the HDM2 protein and rapid proteasomal degradation. Following DNA damage and other stresses, the human p53 *N*-terminal region is phosphorylated. The phosphorylation of p53 disrupts its binding with HDM2, blocks ubiquitination and proteolysis, and results in a rapid increase in p53 protein levels, allowing p53 to enter the nucleus, bind to DNA and induce expression of DNA repair and cell cycle inhibitor genes. Thus, p53 facilitates the repair and survival of damaged cells or eliminates severely damaged cells from the replicative pool to protect the organism. Selenoprotein W (SEPW1) is a highly conserved small thioredoxin-like protein required for cell cycle progression. In a study carried out in breast cancer cells (MCF-7) SEPW1 controlled p21 by modulating levels of the p53 transcription factor [72]. It is well known that metal complexes have been one of the most widely employed anticancer drugs. The complexation of Se compounds with different transition metals as antitumoral agents has been reported in the literature [73]. Later, these authors [74] investigated the mechanism of antiproliferative activity of these compounds and concluded that they induced apoptosis, provoked changes in cell cycle distribution, caspase-3 activation and activation of the p53 tumor suppressor gene family p73. Other organoselenium derivatives, such as PBISe, induced apoptosis in human non-small cell lung cancer with significantly enhanced levels of p53 [75]. On the other hand, SeCys in human breast carcinoma cells (MCF-7) provoked apoptosis with involvement of p53 phosphorylation and ROS generation [76]. In addition, several well-known inorganic forms of Se, such as sodium selenite, implicated to p53 in apoptotic processes *in vitro* against acute promyelocytic leukemia cells (NB4) [77] as well as in prostate cancer cells (LNCaP) [78].

## 6. Apoptosis and Miscellaneous Mechanisms and Structures

It is well known that apoptotic cascades can be triggered by either extrinsic receptor-mediated, intrinsic mitochondria-mediated or endoplasmic reticulum (ER) stress-mediated signaling pathways. Se induces cellular apoptosis, presumably by acting on the functions of many intracellular proteins important for these apoptotic cascades. In addition, depending on the form, Se compounds can target separate pathways and the existence of diverse responses for the same chemical structure suggest several mechanisms of action and targets [79]. In an experiment comparing the apoptotic mechanisms of the Se compounds MeSeCys, SeMet and sodium selenite in four cell lines from human oral squamous cell carcinoma (HSC-3 and HSC-4), human non-small cell lung adenocarcinoma (A549) and human breast cancer (MCF-7), showed a different response based on the both the compound and the cell line used. SeMet increased apoptotic cells in a p53 dependent manner in A549 whereas MeSeCys increased apoptotic cells in HSC-3 with high activities of caspase-3, -8 and -9. On the other hand, sodium selenite and MeSeCys reduced phosphorylated Akt levels [80].

The heteropoly complex (C_2_H_10_N_2_)_5_(NH_4_)_4_H_2_[Se_2_W_10_V_8_O_62_]·9H_2_O (SeWV) exhibited apoptotic response in K562 cells with accumulation of ROS, reduction of pH and mitochondrial membrane potential, significant inhibition of the expression of Bcl-2 and changes in cell cycle phases [81]. The structural modification of SeWV to corresponding (NH_4_)_4_H_4_[Se_2_Mo_2_V_4_O_24_]·7H_2_O (SeMoV), led to a better antitumor activity and its mechanism was attributed partially to apoptosis with elevation of ROS concentration [82].

**Figure 9:**
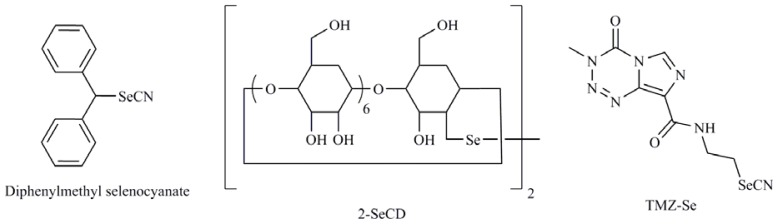
2-Selenium-bridged β-cyclodextrin (2-SeCD), diphenylmethyl selenocyanate, temozolomide-selenium (TMZ-Se).

The organic compound SeMeCys sensitized renal cancer cells through TRAIL mediated downregulation of Bcl-2 [83]. Unfortunately, many cancers develop resistance to TRAIL and for this reason TRAIL sensitising agents are currently being explored. One of the strategies used to achieve this aim was the development of Se formulation as cyclodextrins. So 2-selenium-bridged β-cyclodextrin (2-SeCD) (Figure 9), an organoselenium compound with glutathione peroxidase mimetic activity showed reduction in the viability of MDA-MB-468 and T47D breast carcinoma cells through induction of the expression of TRAIL both *in vitro* and *in vivo* [84].

Another strategy is the incorporation of Se to organic forms, such as bonded to polysaccharide. Shang *et al.* [85] prepared a Se containing polysaccharide (SeGLP-2B-1) from *Ganoderma lucidum* with antiproliferative activity against six cancer cell lines *in vitro*. Later, these authors [86] investigated its antitumor mechanism in a study carried out with MCF-7 cell line. They demonstrated the apoptotic effect of SeGLP-2B-1 with disruption of the mitochondrial membrane potential followed by an increase in the cytochrome c cytosolic levels, and an increase in the activities of caspases 9 and 3. Furthermore, it has been shown that the Se enrichment of broccoli sprout extract enhances its anticancer properties. In a study with human prostate cancer cell line (LNCaP), this extract inhibited cell proliferation with apoptosis induction and downregulation of the Akt/mTOR survival pathway [87]. Similar effects were observed with Se enriched garlic or organoselenium compounds that provided more potent protection against mammary carcinogenesis in rats and greater inhibition of ROS and apoptosis induction in breast cancer cells in culture, than natural garlic or the respective organosulfur analogues [88].

In recent years the combination of Se with widely studied chemotherapeutic agents is being studied. So, genistein-selenium combination (Gn-Se) significantly inhibited growth of LNCaP and PC-3 cells through caspase dependent apoptosis induction with evidence of an alternative non-caspase pathway [89]. On the other hand, the therapeutic efficacy of irinotecan is augmented in combination with MeSeCys probably due to an increase in the intra-tumor concentrations of SN-38, an active metabolite of irinotecan, and significantly increased the apoptosis incidence when compared to irinotecan alone [90]. Also, it was observed that when cyclophosphamide (CP) was administered with diphenylmethyl selenocyanate (Figure 9), the Se derivative restored the levels of antioxidant enzymes system and resulted in significant tumor growth regression along with upregulation of apoptosis and reduction of cellular toxicity of CP simultaneously improving its antitumor efficacy [91]. Other possibility is the rational incorporation of Se into molecules with recognized efficacy in cancer. An example is temozolomide (TMZ) that is widely used in the treatment of human malignancies such as glioma and melanoma. Cheng *et al.* [92] synthesized a new TMZ selenium analog (TMZ-Se) (Figure 9) by introducing of an *N*-ethylselenocyanate extension to the amide functionality in TMZ structure. The introduction of Se into TMZ produced superior cytotoxicity to TMZ in human glioma and melanoma cells. Furthermore, this compound caused a greater DNA damage response, more severe impairment of mTOR signaling, was more apoptogenic than TMZ in tumor cells, and as a novelty induced greater autophagic response.

Moreover, Se is able to revert the carcinogenic effects induced by azoxymethane and dextran sodium sulfate on colon carcinogenesis in a mouse model with high iron diet. In a study carried out with five experimental groups the incidence of colon tumor formation in the high Se diet group was 19% lower than control and the apoptotic index was significantly higher than other groups [93].

## 7. Apoptosis *versus* Autophagy

Autophagy is a type of programmed cell death alternative to apoptosis and recent studies have established that it also plays an important role in cancer biology. However, exactly how autophagy intersects with cancer development, disease progression, and therapeutic response is controversial. The relationship between autophagy and apoptosis is complex and varies with cell types and the specific stress placed upon the cell. While the molecular mechanisms leading to apoptosis have been elucidated to some extent during the past 15 years, autophagic cell death is not well characterized at the molecular level yet. Expanding the knowledge of the molecular cross-talk among pathways that regulate tumor cell death is crucial in guiding the successful design of future anticancer therapeutics. Apoptosis and autophagy can act as partners to induce cell death in a coordinated or cooperative fashion. Autophagy proteins can also play a role in cellular events that occur during apoptosis. For example, Atg5 may be an independent key player in both apoptosis and autophagy. The low levels of Atg5 cleavage product may have significant effects on apoptosis, but not the intact Atg5 that participates in autophagy. Bcl-2 phosphorylation may not only be a mechanism for regulating apoptosis and a mechanism for regulating autophagy, but perhaps also a mechanism for regulating the switch between the two pathways. JNK is able to trigger autophagy by targeting Bcl-2/Bcl-xL proteins and abrogating their binding to Beclin 1. Recently, Beclin 1 has been shown to be among the substrates of death-associated protein kinase (DAPK), a proapoptotic serine/threonine kinase, and its phosphorylation reduces its binding to the Bcl-2 family members, thus suggesting a possible mechanism by which DAPK may also induce autophagy. A complex role in the regulation of autophagy is played by p53, one of the most important tumor suppressor proteins. In fact, p53 regulates autophagy both in a positive and in a negative fashion, depending on its subcellular localization [39]. p53 and damage-regulated autophagy modulator (DRAM) can induce accumulation of autophagosomes. In addition, p53 affects autophagy by modulating signaling through the mTOR nutrient-sensing kinase which controls autophagy at the initiation stage. Inhibition of autophagy leads in most cases to an increased susceptibility to apoptotic stimuli. Moreover, a growing number of proteins that play a negative regulatory role in both events have been identified. Autophagy and apoptosis may be triggered by common upstream signals. Recent reports have shown that Akt inhibits apoptosis by phosphorylation of the Bcl-2 protein family member, Bad, allowing for cell survival. In addition, activation of the PI3K/Akt/mTOR pathway can cause inhibitory effects of Akt on apoptosis and mTOR on autophagy and enhance survival capacity in neoplastic cells. Therefore, it is likely that induction of apoptotic or autophagic cell death may depend on the level of mitochondrial membrane permeabilization. The induction of autophagic cell death may be an ideal approach for cancers that are resistant to apoptosis by anticancer therapies [94,95].

**Figure 10:**
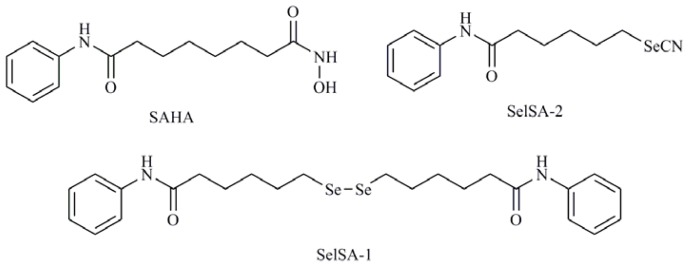
Suberoylanilide hydroxamic acid (SAHA), bis(5-phenylcarbamoylpentyl) diselenide (SelSA-1), phenylcarbamoylpentyl selenocyanide (SelSA-2).

Autophagy has been reported to play a role in the activity of suberoylanilide hydroxamic acid (SAHA) (Figure 10) which has emerged as an effective anticancer agent and is recently approved by the FDA for the treatment of advanced cutaneous T-cell lymphoma [96]. Mechanistically, SAHA regulates autophagy by inducing both LC3 expression transcriptionally and inactivating mTOR, which might be indirect or not at all related to transcription [97]. Hystone deacetylase (HDAC) inhibitors are emerging as potent anticancer agents that can reactivate gene expression and restore the capability of malignant cells to undergo programmed cell death and are able to mediate the induction of both apoptosis and autophagy [94]. Desai *et al.* [98] have synthesized several Se containing analogs of SAHA and have studied its mechanism of action demonstrating that they act as HDAC inhibitors. Among the reported derivatives, bis(5-phenylcarbamoylpentyl) diselenide (SelSA-1) and 5-phenylcarbamoylpentyl selenocyanide (SelSA-2) (Figure 10) are significantly more effective in inducing cytotoxicity (in H460 and H441 cells), in the inhibition of MAPK signaling and in the induction of autophagy in lung cancer cells (A549 cells) than SAHA [99].

## 8. Selenium, Cancer and Cell Death Types: Conclusions, Paradox and Perspectives

Our understanding of the significance of Se in cancer therapy and cell death has expanded greatly in recent years. In this review we have summarized information on more than 100 references, most of which were published in last three years, because our research team had published a review article in 2008 related to Se and apoptosis [100]. This list of compounds and references is by no means exhaustive and merely hints at the hundreds of other citations due to the ever-increasing amount of work carried out in this field. Numerous studies and strategies have been suggested for application of Se in cancer including the utilization of Se in combination with other therapies. For example, Se enhances the antitumor effects of photodynamic therapy (PDT) in TC-1 tumor cells and implanted mice [101]. Also, the modulation of the GSH system mediated by the expression of different factors such as Trx1/p38(MAPK)p53 signaling pathway [102] or a decrease in the expression of Se binding protein 1 (SBPI) that could be a novel biomarker for predicting prognosis and guiding personalized therapeutic strategies, especially in patients with advanced hepatocellular carcinoma (HCC) [103]. One of the problems of the use of Se is the narrow margin between toxic amounts and the amounts needed for dietary requirements or therapeutic effects. In order to solve these limitations Nano-Se had significantly lower toxicity, without compromising the important therapeutic capacities [104]. On the other hand, the practical use of Se and seleno compounds, such as SeO_3_^2−^, MSA, SeMet, and selenocystine is extendable to the treatment of illness caused by parasites derived from *Plasmodium* by inducing apoptosis-like cell death with involvement of ROS [105]. It is interesting to point out that the effectiveness of certain molecules as both anticancer drugs and antiprotozoal agents suggested that this class of compounds and their derivatives might be useful for both the diseases [106–108].

In spite of the aforementioned and of the importance of Se in control disturbances in the redox state of sick cells for therapeutic advantage in cancer treatment, there are evidences from numerous clinical and experimental studies that have shown that the antioxidant activities of similar Se-containing compounds are not identical, suggesting that each compound must be examined individually for its antioxidant behavior [109,110]. For these reasons, there are contradictory literature reports of their efficacy. Brozmanova *et al.* [7] explained the broad interest to exploit the positive effects of Se on human health and cancer therapy and have studied the negative effects such as toxicity and DNA damage induction resulting from high Se intake. These results have been corroborated by Lee *et al.* [111]. An overdose of Se generates oxygen radicals and leads to apoptotic cell death by inducing oxidation and cross linking of protein thiol groups essential for cell survival. Based on the results from the biological studies it is evident that Se seems to have both harmful and beneficial attributes and by a simple modification in the structure, new potent analogues with high therapeutic index and the desired anticancer and apoptotic activities can be generated. Further studies are warranted to pinpoint the structural requirements of Se compounds necessary for maximum efficacy and to explore the true potential of this promising dietary trace element.

## Figures and Tables

**Table 1:** Classification of Se compounds based on structural features.

**Selenoaminoacids**
Selenomethionine (SeMet); Methylselenocysteine (MeSeCys); Selenocystamine; Selenocysteine (SeCys)
**Se-heterocyclic compounds**
1,3-Selenazolin-4-one derivatives; 2,5-Bis(5-hydroxymethyl-2-selenienyl)-3-hydroxymethyl-*N*-methylpyrrole (D-501036); 2-Phenyl-1,2-benzisoselenazol-3(2*H*)-one (ebselen); 1,2-[Bis(1,2-benzisoselenazolone-3-(2*H*)-ketone)]ethane (BBSKE); 2-Buthylselenazolidine-4-(*R*)-carboxylic acid (BSCA); 2-Cyclohexylselenazolidine-4-(*R*)-carboxylic acid (ChSCA)
**Selenocyanates**
1-4-phenylenebis(methylene)selenocyanate (*p*-XSC); Isatin analogs; Diphenylmethylselenocyanate; TMZ-Se; 5-phenylcarbamoylpentyl selenocyanide (SelSA-2)
**Isoselenocyanates**
4-Phenylbutylisoselenocyanate (ISC-4)
**Diselenides**
Diselenodipropionic acid (DSePA); Bis(4-aminophenyl)diselenide; 2-Selenium-bridged β-cyclodextrin (2-SeCD); Bis(5-phenylcarbamoylpentyl) diselenide (SelSA-1)
**Selenides**
Se,Se′-1,4-phenylenebis(1,2-ethanediyl)bisisoselenourea (PBISe); Methylimidoselenocarbamates; 5-Phenylselenyl-methyl-2′-deoxyuridine (PhSe-T); 5-Methylselenyl-methyl-2′-deoxyuridine (MeSe-T); β-selenium amine derivatives
**Se(IV) compounds**
Sodium selenite (Na_2_SeO_3_); Methylseleninic acid (MSA); Selenous acid; Selenium dioxide (SeO_2_)
